# Telocytes inhibited inflammatory factor expression and enhanced cell migration in LPS-induced skin wound healing models in vitro and in vivo

**DOI:** 10.1186/s12967-020-02217-y

**Published:** 2020-02-06

**Authors:** Lu Wang, Dongli Song, Chuanyuan Wei, Cheng Chen, Yanwen Yang, Xinyi Deng, Jianying Gu

**Affiliations:** 1 Department of Plastic Surgery, Zhongshan Hospital, Fudan University, Shanghai, 200032 People’s Republic of China; 2 Zhongshan Hospital Institute for Clinical Science, Shanghai Institute of Clinical Bioinformatics, Shanghai Engineering Research for AI Technology for Cardiopulmonary Diseases, Shanghai Medical College, Fudan University, Shanghai, China

**Keywords:** Telocyte, Wound healing, LPS, Proliferation, Apoptosis

## Abstract

**Background:**

Cell proliferation and death are key components of wound healing and tissue repair. Telocytes (TCs) represent a newly discovered cell type that can protect tissue from acute injury via cell–cell communication with adjacent cells. The aim of this study was to use a mouse model of skin wound healing and lipopolysaccharide (LPS)-induced cell injury to evaluate the effects of TCs on skin wound healing in vivo and in vitro.

**Material/methods:**

Immunohistochemical staining was performed to evaluate the alteration of TCs in tissues from normal and chronic wound patients. Then, a male C57BL/6 mouse wound model of the back was established. The mice were divided randomly into three groups, and wound healing was estimated according to the wound healing rate and histology. An LPS-induced co-culture model of a mouse lung telocyte cell line (TCs) with human keratinocyte (HaCaT), human dermal microvascular endothelial cell (HDMEC) or murine fibroblast (L929) cell lines was established to analyse the effects of TCs on constitutive cell types of the skin. Cell proliferation, migration and apoptosis were examined, and reactive oxygen species (ROS) and inflammatory factors in HaCaT cells, HDMECs, and L929 cells were detected to study the mechanisms involved in TC protection in skin wounds.

**Results:**

TCs were significantly increased in tissues from chronic wound patients compared with healthy controls. Wound healing was significantly improved in wound mouse models treated with exogenous TCs compared with LPS-induced models. TCs reversed the LPS-induced inhibition of HaCaT cells and HDMECs and reduced the LPS-induced apoptosis of HaCaT cells and the death ratios of HDMECs and L929 cells. TCs reversed LPS-induced ROS in HDMECs and L929 cells and decreased inflammatory factor mRNA levels in HaCaT cells, HDMECs and L929 cells.

**Conclusions:**

TCs reduce wound healing delay, and inflammatory responses caused by LPS might be mediated by inflammatory inhibition, thus restricting apoptosis and promoting migration of the main component cell types in the skin.

## Introduction

Chronic wounds are an intractable clinical problem. Although there have already been many management and treatment strategies, treatment remains a major problem since chronic wounds are apt to relapse. Understanding the mechanisms of chronic wounds could provide an opportunity to search for effective methods to treat chronic wounds. The process of wound healing is complex and coherent and involves four stages: inflammation, granulation tissue formation, re-epithelialization, and shaping after wound healing [1]. During these stages, angiogenesis is essential for wound repair, and the proliferation and migration of keratinocytes and fibroblasts are key points in re-epithelialization [2–4]. Providing the microenvironment for cell migration, proliferation and apoptosis prevention should be an effective method for the repair of wounds.

Telocytes (TCs) represent a newly discovered interstitial cell type that was found by the Popescu group, and they are widely distributed in the tissues and organs of the body, including the heart, lungs, kidneys, liver and other tissues, even in skin [5]. TCs are distinguished from other interstitial cell types, including stem cells and fibroblasts, by protein profiles and gene profiles [6]. Many studies have found that TCs can exert a substantial impact on regeneration and repair, for example, reducing myocardial infarction and acute lung injury [7]. TCs can affect other adjacent cells via direct connection or indirect modes by producing and releasing materials and molecules, including extracellular vesicles, and they are particularly involved in cell-to-cell communication [8]. Recently, studies have demonstrated that TCs exist in skin tissues according to focused ion beam scanning electron microscopy (FIB-SEM) tomography and with the establishment of the 3D reconstruction of dermal TCs [9]. Song et al. recently established a mouse TC cell line (TCs) and demonstrated the maintenance of behavioural morphology and biological characteristics for 50 generations, which provided further patterns for the TC study [10]. However, whether TCs can promote skin wound healing as well as the mechanisms involved in this process remain unclear.

To investigate whether TCs play roles in cutaneous wound healing, immunohistochemical staining was first conducted to detect the distribution of TCs in tissues from normal and chronic wound patients. And the results showed that PDGFRα^+^ TCs accumulated in the dermis of chronic wound tissue. Although chronic wounds can be caused by many kinds of reasons, such as venous hypertension/congestion, arterial insufficiency, prolonged unrelieved pressure or diabetes, they experience a common pathophysiological process: excessive inflammation. Since bacterial biofilms contained LPS is a major impediment to the inflammation of wound healing, LPS-induced male C57BL/6 mouse full-thickness cutaneous wound model was established [11]. The effect of TCs on wound healing was estimated by gross observation and histology. In order to find out the mainly cell type or cell types as the recipient cells downstream of TCs cell–cell communication in LPS induced skin wound, co-culture models of human keratinocyte (HaCaT), human dermal microvascular endothelial cell (HDMEC) [12] or murine fibroblast (L929) cell lines [13] with TCs were established. Cell proliferation, migration and apoptosis, and ROS and inflammatory factors were examined in HaCaTs, HDMECs, and L929 cell lines were detected to study the potential mechanisms involved in TCs protection in the skin wound healing process.

## Materials and methods

### Patients

Our study enrolled three patients who suffered from diabetic foot, venous ulcers and pressure ulcers. Three normal control tissues were obtained from three patients, one of whom suffered from a benign nevus and 2 from lipoma. All chronic wound patients were subjected to debridement, and all control patients underwent lumpectomy. All patients signed an informed consent form, and ethical approval was provided by the Ethics Committee of Zhongshan Hospital.

### Animals and experimental groups

Animal studies were performed according to the national guide for the care and use of laboratory animals. Ethical approval was acquired from the Ethics Committee of Zhongshan Hospital. Twenty-four male C57BL/6 mice, aged 8–10 weeks, were used in the experiment. The mice were divided into four groups, and each mouse was anaesthetized with 1% pentobarbital sodium (50 mg/kg) and received a 6-mm skin wound on the back via scissors; after that, different groups received different treatments as follows: (i) the vehicle group (n = 12) received local injections of 0.2 ml phosphate-buffered saline (PBS); (ii) the LPS-induced group (n = 12) was intradermally injected with 10 μg/ml LPS (Sigma, Saint Louis, USA) dissolved in 0.2 ml PBS; (iii) the TCs treated group (n = 12) was intradermally injected with 1 × 10^6^ TCs suspended in 0.2 ml PBS; and (iv) LPS-induced and TCs treated combined group (n = 12) was intradermally injected with 10 μg/ml LPS dissolved in 0.1 ml PBS and then treated with 1 × 10^6^ TCs suspended in 0.1 ml PBS local injection. Different reagents and cells were intradermally injected around each wound, and injections were given every other day. Three and seven days after wounding, the tissues were excised for histological analysis. The remainder of the mice were used to evaluate the speed of wound healing. The wound was photographed with a camera after surgery on days 0, 2, 4, 6 and 8. Then, the photos were analysed by using ImageJ software. All the photos were taken accompanied with a ruler that served as the standard. The wound healing rate (percentage) was calculated as follows: the area of the actual wound/the area of the original wound × 100%.

### Cell culture

Mouse TCs were kindly provided by Dr. Dongli Song (Zhongshan Hospital Institute for Clinical Science, Fudan University, Shanghai, China). This TCs were isolated from mouse and transfected with SV40 large and small T antigen, and identified with telopodes (Tps) and the expression of ckit, CD34, vimentin and PDGFRα from generation 5 to generation 50 [10]. Human dermal endothelial cells (HDMECs), human immortalized keratinocyte (HaCaT) cell line, and L929 mouse fibroblast cell line were purchased from the Cell Bank of the Chinese Academy of Sciences (Shanghai, China), and they were cultivated in the recommended media and conditions. To detect the effect of TCs on HaCaT cells, HDMECs and L929 cells with the stimulated by LPS, the three kinds of cells were divided into four groups as follows: (i) the vehicle group, cells treated with serum-free medium; (ii) the LPS-induced group, cells treated with serum-free medium containing 1 μg/ml LPS; (iii) the TCs-cocultured group, HaCaT/HDMEC/L929 cell lines cultured in plates with TCs in transwell co-culture system; and (iv) the LPS-induced TCs-cocultured group: HaCaT/HDMEC/L929 cell lines co-cultured with TCs in transwell system and stimulated with LPS. Cells were seeded in well plates with a density of 10^5^ cells/ml, and the medium was replaced with serum-free medium 24 h before LPS stimulation.

### Histology and immunohistochemical staining

Histological sections were prepared from tissues excised 3 and 7 days after wounding, fixed in 10% buffered formalin and embedded in paraffin. The 5-μm sections were stained with haematoxylin and eosin (H&E) to detect the infiltration of inflammatory cells.

Recent studies demonstrated that PDGFRα was a credible marker for TCs identification [14]. For the immunohistochemical staining, the slides were deparaffinized with xylene and rehydrated with a gradient of ethyl alcohol. After antigen retrieval with 0.3% trypsin and after blocking endogenous peroxidases with 3% H_2_O_2_ and blocking nonspecific antigens with blocking solution (KeyGEN, Jiangsu, China), the sections were incubated with primary antibodies for PDGFRα at 1:100 (ab90967; Abcam, Cambridge, UK) overnight at 4 °C, and then the immunohistochemistry kits (KeyGEN, Jiangsu, China) were used for 1 h. The sections were observed at × 40 and × 200 magnification, and images were captured using a standard Olympus microscope (Olympus, Tokyo, Japan).

### Transmission electron microscopy (TEM)

Fresh TCs culture samples were collected in PBS at pH 7.4 and prepared for TEM as previously described [15]. Grids were observed in JEOL JEM-1230 (Tokyo, Japan) electron microscope. Digital electron micrographs (2048 × 2048 pixels, 4 MB, and uncompressed grayscale Tiff files) were obtained with a high-resolution digital camera Olympus MegaView III connected to the electron microscope (Olympus, Soft Imaging System GmbH, Münster, Germany).

### Immunofluorescent staining

Immunofluorescent staining for CD34/Vimentin/PDGFα was used as previously reported for TCs identification. In brief, TCs were seeded on glass bottom cell culture dishes with 15 mm diameter glass (NEST, Nanjing, China) overnight. Then, cells were fixed in 4% paraformaldehyde containing 0.05% Triton-X-100 for 20 min and the dishes were washed for three times with 1 × PBS before blocking in 5% Bovine serum albumin (BSA) for 1 h. After incubated with mouse anti-CD34 antibody, goat anti-vimentin antibody or rat anti- PDGFRα antibody (1:200 dilution; Abcam, Cambridge, UK) diluted in 1% bovine serum albumin (BSA) in PBS overnight at 4 °C, the dishes were washing in PBS for three times. Cells were incubated with APC conjugated anti-mouse secondary antibodies, PE conjugated anti-rat secondary antibodies and FITC conjugated anti-goat secondary antibodies (1:200 dilution; Jackson ImmunoResearch, USA) for other 1 h at 4 °C in dark. The nuclear were marked with DAPI according to the manufacture (KeyGEN BioTECH, Nanjing, China). TCs were observed and recorded using Olympus FV3000 Confocal Laser Scanning Microscope (DSS Imagetech Pvt. Ltd, New Delhi, India).

### Live measurement of cell bio-behaviours

To detect the bio-behaviours of HaCaT cells, HDMECs, and L929 cells, a Cell-IQ cell culturing platform was used (ChipMan Technologies, Tampere, Finland) and equipped with a microscope (Nikon CFI Achromat phase contrast objective with 10 magnification) and a camera 20. Bio-behaviours included cell proliferation and cell movement. Images were captured at 2-h intervals for 48 h. Analysis was conducted by using image software (Cell-IQ Imagen v2.9.5c, McMaster Biophotonics Facility, Hamilton, ON, Canada). Bio-behaviours of TCs induced by LPS were recorded by Cell-IQ, as well.

### Apoptosis analysis

After processing the cells as described above, the cells were collected in six-well plates, washed with PBS, and then incubated in annexin V conjugated with FITC and pidium iodide (PI) (KeyGEN, Jiangsu, China) according to the manufacturer’s instructions. After staining, the cells were analysed with a flow cytometer (FACS Aria II, Becton, Dickinson and Company, NJ, USA), and the results were analysed by using FlowJo v10.0.7 software.

### Determination of ROS

After processing the cells as described above, the cells were collected and dispensed in serum-free medium containing 10 μmol/L DCFH-DA (KeyGEN, Jiangsu, China), incubated at 37 °C for 20 min, turned upside down every 5 min, and washed three times with serum-free medium. The cells were analysed with a flow cytometer (FACS Aria II, Becton, Dickinson and Company, NJ, USA), and the results were analysed by using FlowJo v10.0.7 software.

### Real-time reverse transcription PCR (RT-PCR) analysis

Total RNA was extracted using TRIzol reagent and reverse-transcribed using the cDNA synthesis kit (Takara, Dalian, China); SYBR Green PCR Master Mix (Takara, Dalian, China); and a real-time PCR system (ABI7500, Applied Biosystems). The primer sequences (5′–3′) used were as follows: forward: 5′-GCGGCATCCAGCTACGAATCTC-3′, reverse: 5′-AACCAGCATCTTCCTCAGCTTGTC-3′ for human IL-1b (interleukin-1b); forward: 5′- CACTGGTCTTTTGGAGTTTGAG-3′, reverse: 5′-GGACTTTTGTACTCATCTGCAC-3′ for human IL-6 (interleukin-6); forward: 5′-GTGATGGCTGAACTGTCGCC-3′, reverse: 5′-CTGGGATGCTCTTCGACCTC-3′ for human IFN-γ (interferon-γ); forward: 5′-CCAATGGCGTGGAGCTGAGAG-3′, reverse: 5′-TCTGGTAGGAGACGGCGATGC-3′ for human TNF-α (tumour necrosis factor α); forward: 5′-TCCTGGTGCTCCTGGTGCTG-3′, reverse: 5′-CTGCCTGTCGGTGAGATTGGTTC-3′ for human MMP9 (matrix metalloproteinase 9); forward: 5′-GACATGGTGGTCGGCTTCGC-3′, reverse: 5′-CGCCTCTGTCATTCGTGCTTCC-3′ for human NFkB (nuclear factor kappa B); forward: 5′-CCTGGCACCCAGCACAAT-3′, reverse: 5′-GGGCCGGACTCGTCATAC for human β-Actin; forward: 5′-TCGCAGCAGCACATCAACAAGAG-3′ reverse: 5′-TGCTCATGTCCTCATCCTGGAAGG-3′ for mouse IL-1b; forward: 5′-ACTTCCATCCAGTTGCCTTCTTGG-3′, reverse: 5′-TTAAGCCTCCGACTTGTGAAGTGG-3′ for mouse IL-6; forward: 5′-CAGGCCATCAGCAACAACATAAGC-3′, reverse: 5′-AGCTGGTGGACCACTCGGATG-3′ for mouse IFN-γ; forward: 5′-GCGACGTGGAACTGGCAGAAG-3′ reverse: 5′-GCCACAAGCAGGAATGAGAAGAGG-3′ for mouse TNF-α; forward: 5′-GGAGCACGGCAACGGAGAAG-3′ reverse: 5′-CCTGGTCATAGTTGGCTGTGGTG-3′ for mouse MMP9; forward: 5′-ATCACCAACCAGCCAGGAATTGC-3′, reverse: 5′-CTGCGTCAAGACTGCTACACTGG-3′ for mouse NFkB; forward: 5′-TGGTCCTGCTGCTCGTCTTGG-3′, reverse: 5′-GTCCGCTGCTGCTCACACTTC-3′ for mouse EGF. The fold change in the relative gene expression to the control levels was determined using the standard 2^−ΔΔCt^ method.

### Statistical analysis

Data are presented as the mean ± standard error of the mean (SEM). Statistical analysis was performed using by one-way analysis of variance (ANOVA) and Tukey’s multiple comparisons test. P < 0.05 was considered statistically significant. All statistical analysis was performed using SPSS Statistics 20 (IBM, Chicago, USA).

## Results

### Accumulation of TCs in chronic wound tissues

**Fig. 1:**
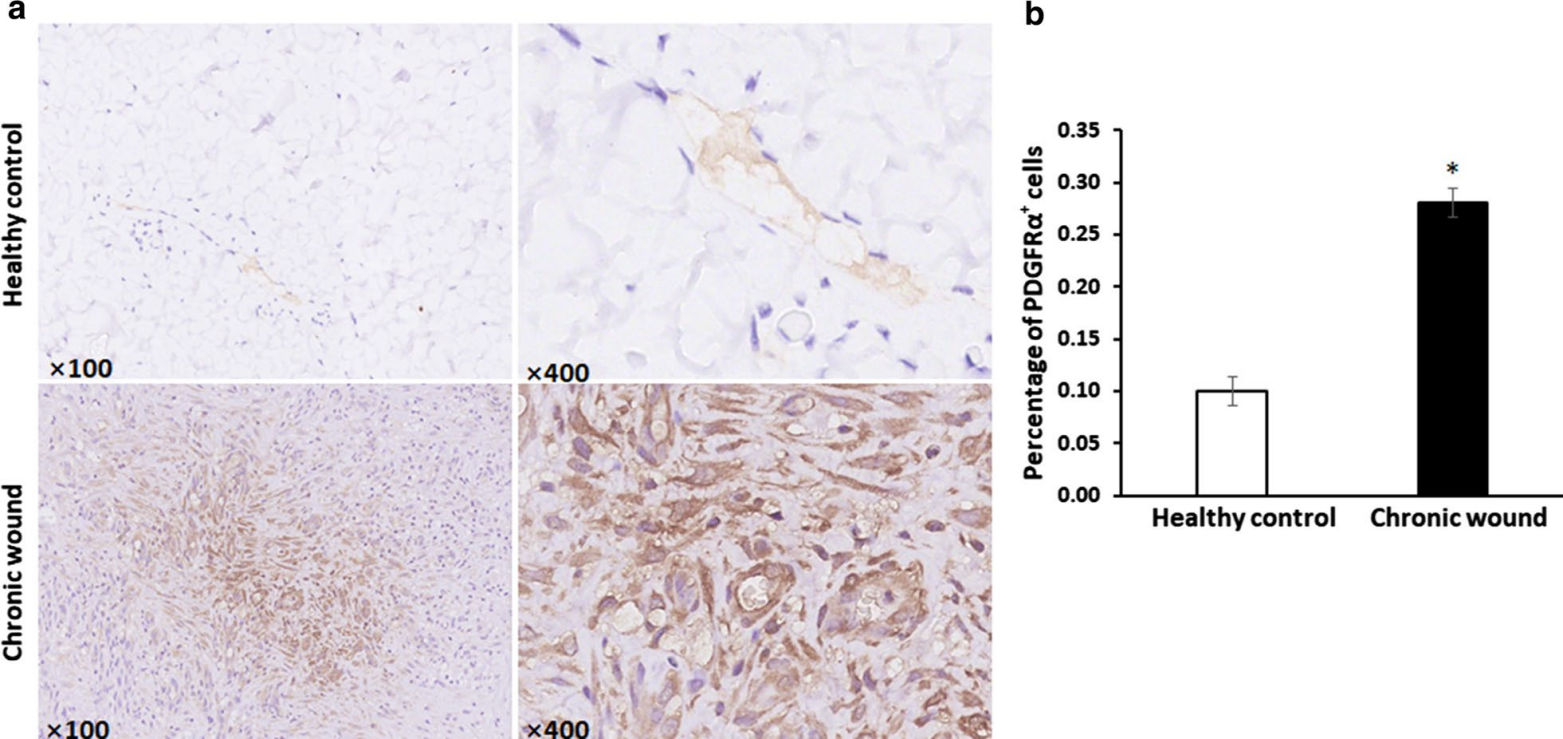
Accumulation of TCs in chronic wounds tissues. **a** Telocytes stained with PDGFRα in granulation tissue from chronic wound patients and in the dermis of skin. **b** The comparison of percentage of PDGFRα^+^ cells in healthy control and chronic wound. N = 3, ^*^P < 0.05 compared with healthy control. Data are expressed as the mean ± SEM

Sections stained with anti-PDGFRα indicated that TCs were located in the papillary dermis, and revealed the distribution of TCs in granulation tissues from chronic wound patients. In the dermis of the skin, the quantities of TCs in chronic wounds significantly increased compared with that in the normal skin (P < 0.05), which demonstrated that TCs accumulated in the chronic wounding process (Fig. 1).

### TCs were identified with telopodes and typical markers positive expression

**Fig. 2:**
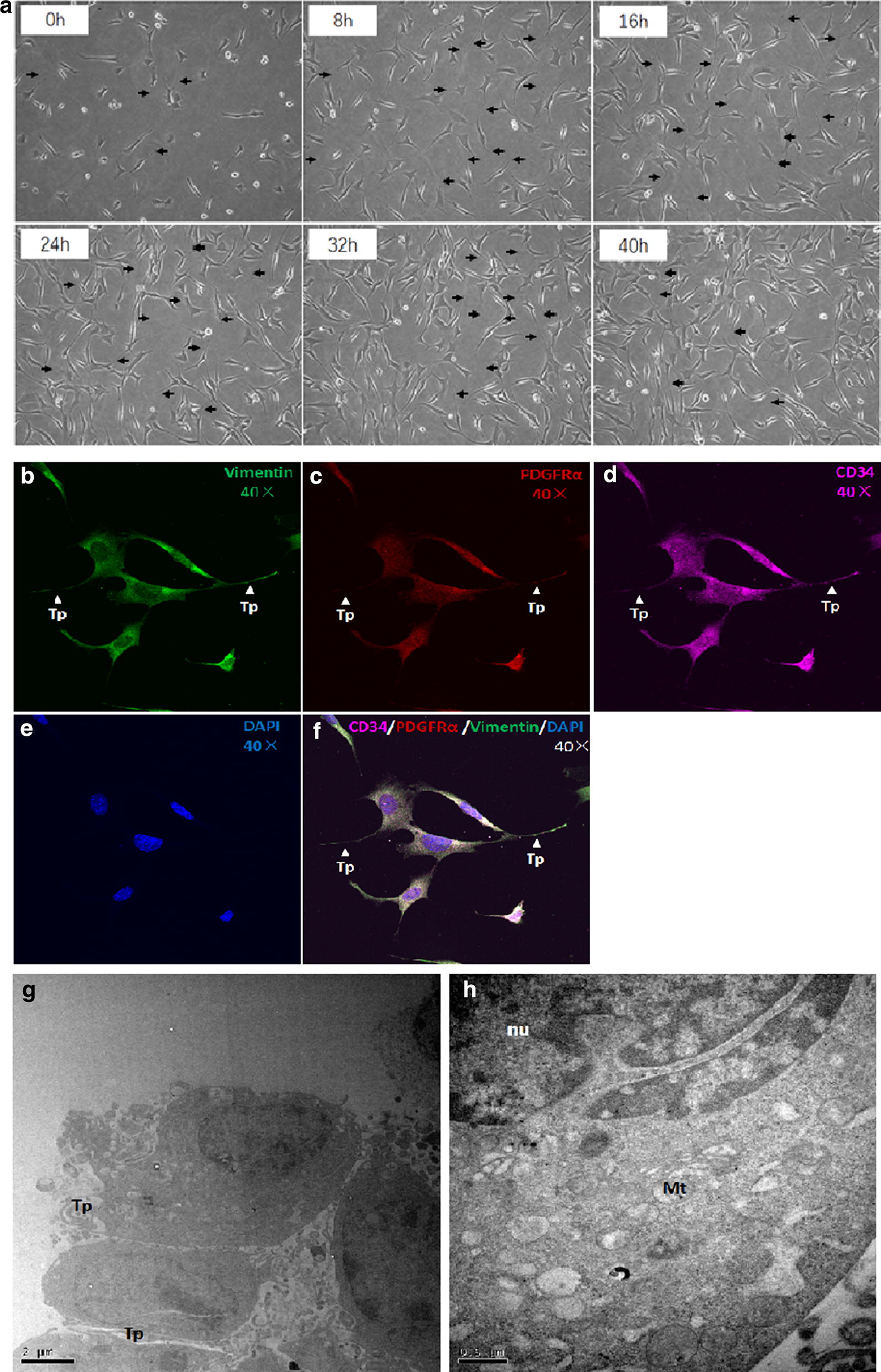
TCs were identified with morphology observation and CD34/PDGFRα/vimentin positive in culture. **a** Representative photos of TCs cultured for 0 h to 48 h captured by Cell-IQ, respectively. Original magnification ×100. Immunofluorescence labelling for vimentin (**b**, green), PDGFRα (**c**, red) and CD34 (**d**, purple) with DAPI (**e**, blue) counterstain for nuclei. TCs are CD34, PDGFRα and vimentin positive (**f**). Arrows and arrow heads show typical TCs with long and thin telopodes (Tp) with dilations. Original magnification ×400. The ultrastructure of mouse TCs are shown in **g** and **h**. Tp: telopode; nu: nucleolus; Mt: mitochondria

Mouse lung TCs were successfully isolated and constructed as a cell line as previously reported. In order to confirm these TCs, we first identified them with the expression of markers typical of TCs. These TCs have special characteristics including relatively small cell body and very long and thin Tps with lots of dilations (Fig. 2a). To further confirm that the cells were TCs, triple immunofluorescent staining for CD34/PDGFRα/vimentin was used. We found that these cells were triple positive for CD34/PDGFRα/vimentin (Fig. 2b–f), indicating that these cells were TCs. TCs morphology were then observed and recorded using Cell-IQ with LPS treated for 40 h. TCs ultrastructure were observed and recorded as well (Fig. 2g).

### Local injection of TCs alleviated inflammatory cell infiltration in LPS-induced wounds

**Fig. 3:**
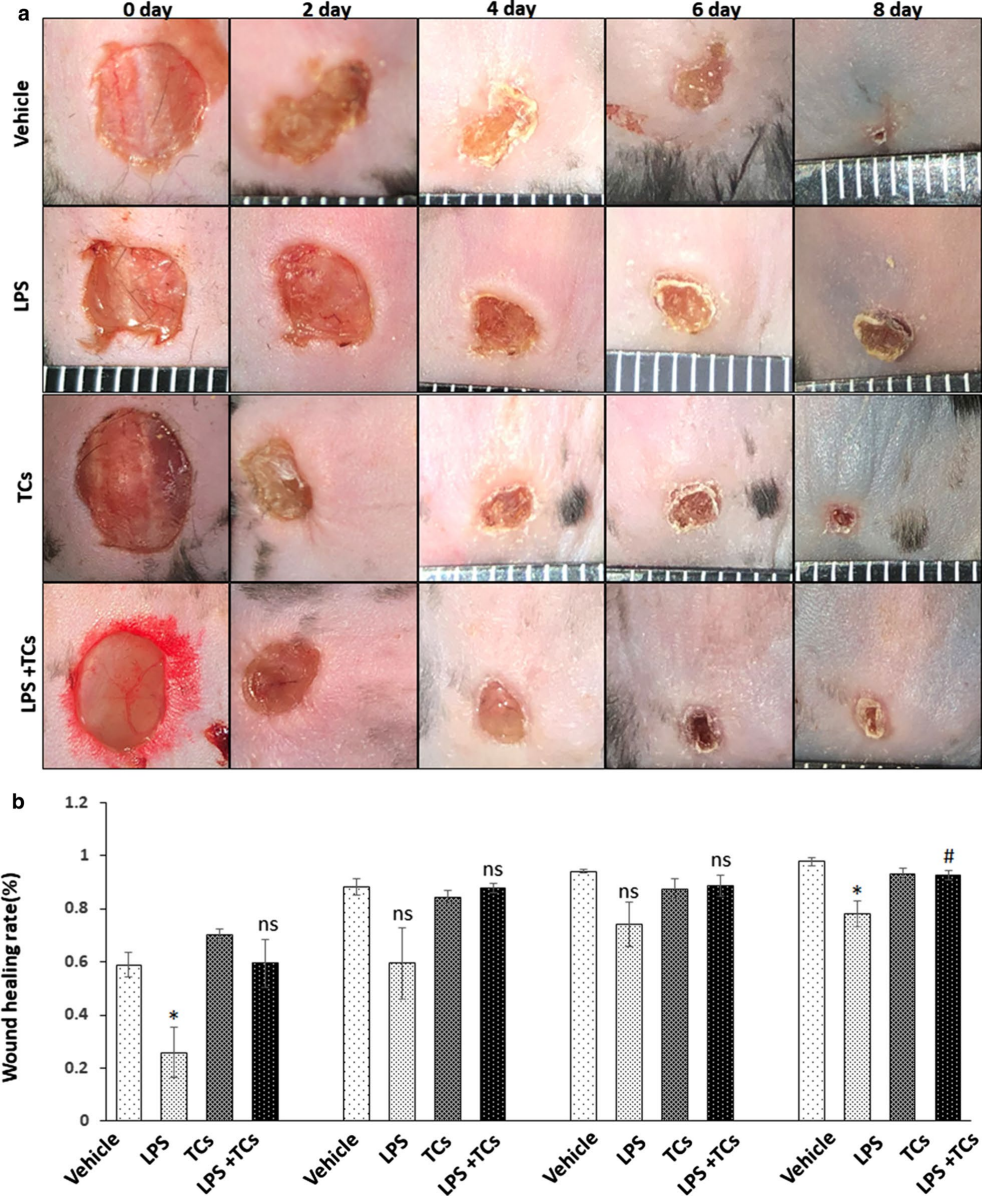
TCs reverse the LPS-induced wound healing delay in mice. **a** General picture of vehicle, LPS-induced, TCs and LPS combined TCs treatment (n = 6). **b** The wound healing rate of vehicle, LPS-induced, TCs and LPS combined TCs treatment in day 0, 2, 4, 8. ^*^P < 0.05 compared with vehicle; #*P* < 0.05 compared with LPS treated group; ^ns^*P* > 0.05, LPS treated group compared with vehicle or TCs-cocultured group compared with LPS-induced group. Data are expressed as the mean ± SEM

**Fig. 4:**
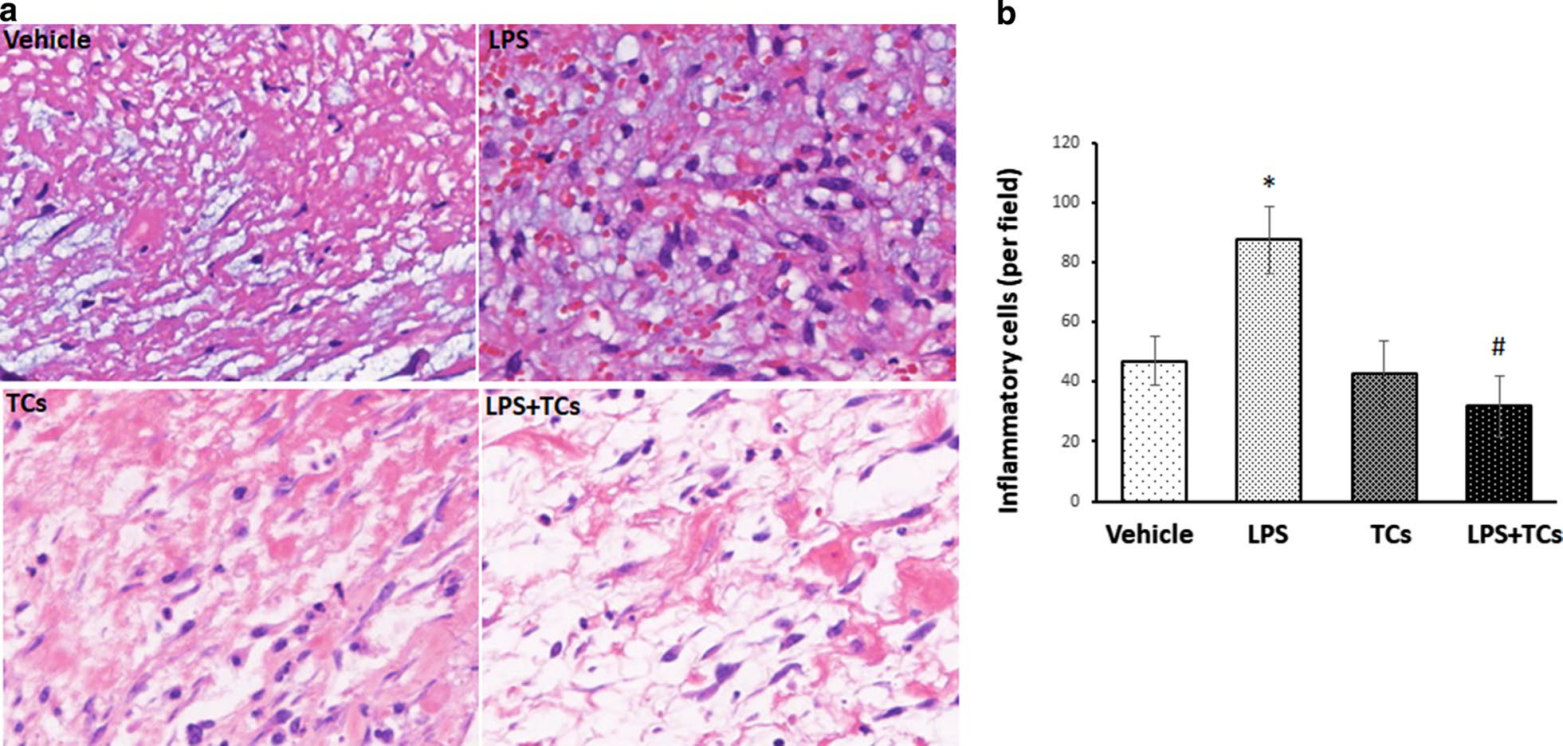
TCs alleviate the inflammation in LPS-induced wound in mice. **a** Inflammatory cells infiltration in wound was detected by histological analysis. **b** The comparison of percentage of inflammatory cells in vehicle, LPS-induced, TCs and LPS combined TCs treatment in day 3. ^*^P < 0.05 compared with vehicle; ^#^*P* < 0.05 compared with LPS treated group; ^ns^*P* > 0.05, LPS treated group compared with vehicle or TCs-cocultured group compared with LPS-induced group. Data are expressed as the mean ± SEM

To investigate the effect of TCs on wound healing, mouse skin wounds were treated with LPS and injected with TCs locally, and observed for 8 days. The results showed that wound healing rate was significantly delayed in the LPS group compared with that of the control on day 2 and day 8 (P < 0.05) (n = 6) (Fig. 3). The rate in the LPS-induced and TCs treatment group was significantly increased compared with that of LPS-induced group (P < 0.05) (n = 6). Histological analysis showed that inflammatory cell infiltration was increased in the granulation tissues of the LPS-stimulated group, while TC injection relieved inflammatory cell infiltration (Fig. 4).

### TCs reversed the LPS-induced inhibition of proliferation and migration in HaCaT cells, HDMECs and L929 cells

**Fig. 5:**
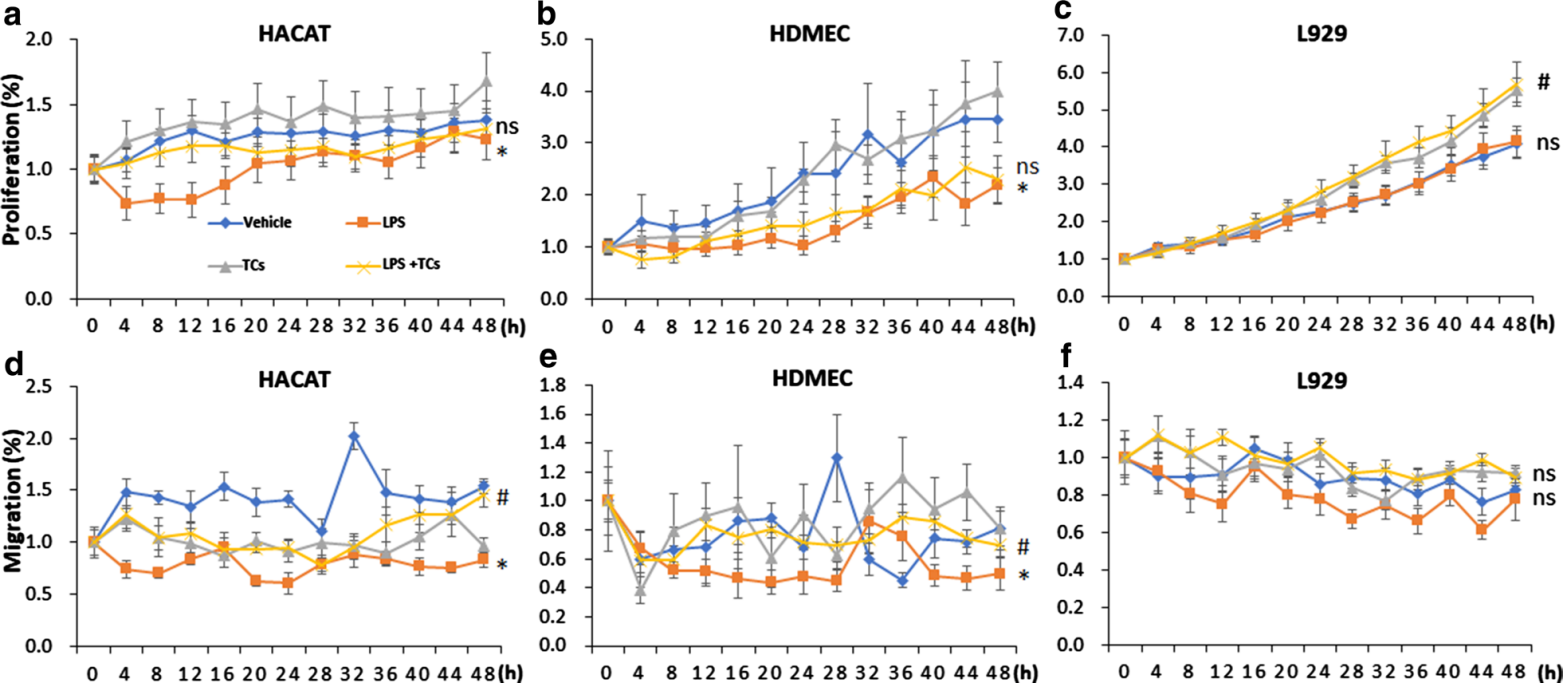
Cell proliferation and cell migration analysis of LPS stimulated HaCaTs, HDMECs and L929 alone or co-cultured with TCs by Cell-IQ. **a**, **d** Cell proliferation and cell migration analysis of LPS stimulated HACAT alone or co-cultured with TCs by cell-IQ. **b**, **e** Cell proliferation and cell migration analysis of LPS stimulated HDMEC alone or co-cultured with TCs by Cell-IQ. **c**, **f** Cell proliferation and cell migration analysis of LPS stimulated L929 alone or co-cultured with TCs by Cell-IQ. N = 6–8, ^*^P < 0.05 compared with vehicle; ^#^*P* < 0.05 compared with LPS treated group; ^ns^*P* > 0.05, LPS treated group compared with vehicle or TCs-cocultured group compared with LPS-induced group. Data are expressed as the mean ± SEM

In order to study the mechanisms of TCs on incisional wound repair, we cultured HaCaT cells, HDMEC cells or L929 cells with TCs in a transwell system and/or with LPS treatment. The results showed that the proliferation of HaCaT cells was significantly decreased with LPS treatment for 20 h, while co-culture with TCs could reverse the anti-proliferation induced by LPS. LPS inhibited the migration of HaCaT cells in 48 h, and TCs ameliorated the inhibitory effect from treatment for 36 h (Fig. 5a, d). LPS inhibited the proliferation of HDMECs, and TCs co-culture did not improve the proliferation of HDMECs with LPS-induced. LPS inhibited the migration of HDMECs in the first 30 h, and TCs partially reversed the suppression of HDMECs migration by LPS from 12 to 18 h (Fig. 5b, e). A total of 1 μg/ml LPS did not inhibit the proliferation of L929 cells; however, TCs improved L929 cell proliferation rates when stimulated with LPS. LPS did not inhibit the migration of L929 cells, and TCs had no effect on the migration of L929 cells with LPS-induced (Fig. 5c, f). Bio-behaviours of TCs induced by LPS were recorded by Cell-IQ are shown in Fig. 2.

### TCs reversed LPS-induced apoptosis on HaCaT cells, HDMECs and L929 cells

**Fig. 6:**
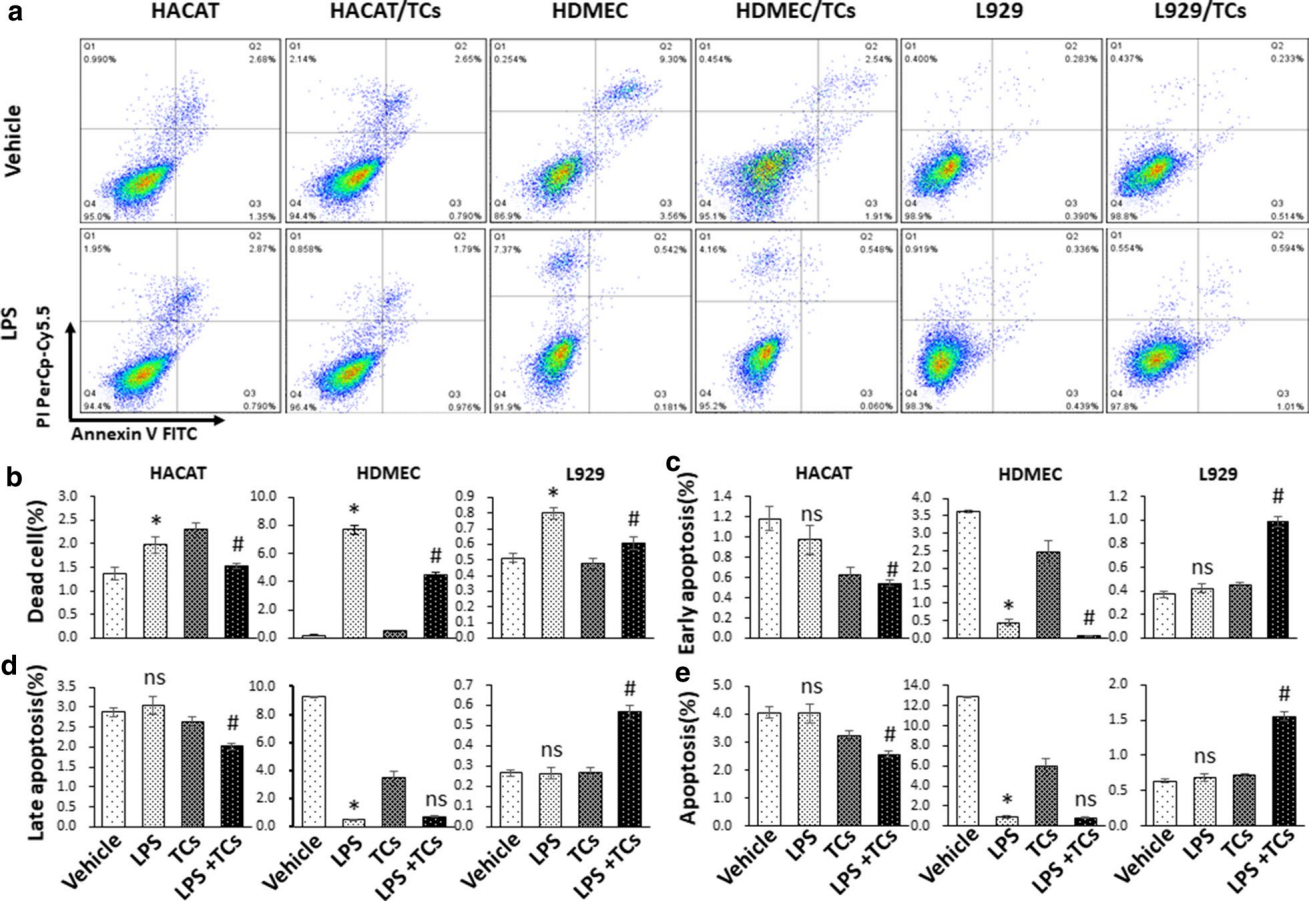
Cell death and apoptosis analysis of LPS stimulated HaCaTs, HDMECs and L929 alone or co-cultured with TCs by Annexin V -FITC/PI double staining in 24 h. **a** Representative pictures of cell apoptosis phases for LPS stimulated HaCaTs, HDMECs and L929 alone or co-cultured with TCs. **b**–**e** The comparison of death, early apoptosis, late apoptosis, and whole apoptosis in LPS stimulated HaCaTs, HDMECs and L929 alone or co-cultured with TCs. ^*^P < 0.05 compared with vehicle; ^#^*P* < 0.05 compared with LPS treated group; ^ns^*P* > 0.05, LPS treated group compared with vehicle or TCs-cocultured group compared with LPS-induced group. Data are expressed as the mean ± SEM

**Fig. 7:**
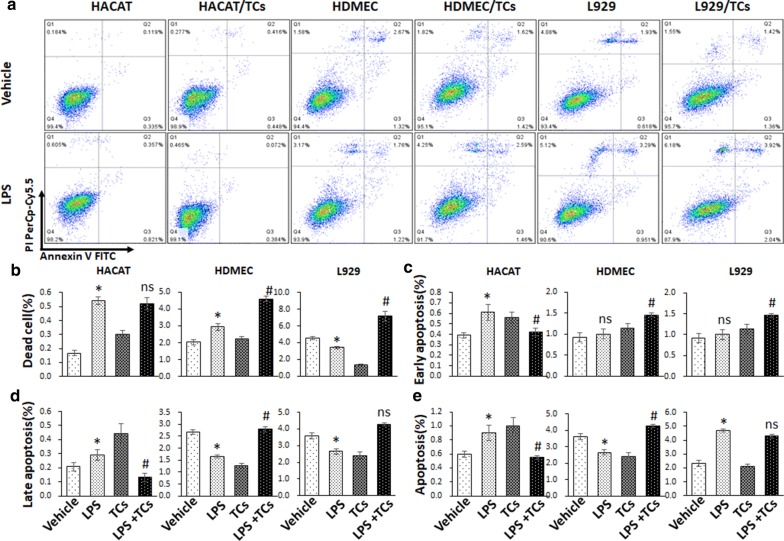
Cell death and apoptosis analysis of LPS stimulated HaCaTs, HDMECs and L929 alone or co-cultured with TCs by Annexin V -FITC/PI double staining in 48 h. **a** Representative pictures of cell apoptosis phases for LPS stimulated HaCaTs, HDMECs and L929 alone or co-cultured with TCs. **b**–**e** The comparison of death, early apoptosis, late apoptosis, and whole apoptosis in LPS stimulated HaCaTs, HDMECs and L929 alone or co-cultured with TCs. ^*^P < 0.05 compared with vehicle; ^#^*P* < 0.05 compared with LPS treated group; ^ns^*P* > 0.05, LPS treated group compared with vehicle or TCs-cocultured group compared with LPS-induced group. Data are expressed as the mean ± SEM

To further confirm the effect of TCs on apoptosis, annexin V and PI staining were used for apoptosis analysis in cells co-cultured with TCs and treated with LPS for 24 h and 48 h. The death ratios in LPS-induced HaCaT cells was significantly increased compared with that of vehicle group, while the TCs-coculture decreased HaCaT cells death ratio after LPS treated. TCs-coculture reduced the ratios of late apoptosis, early apoptosis and whole apoptosis in LPS-induced HaCaT cells (Fig. 6). The ratios of death, late apoptosis, early apoptosis and whole apoptosis in LPS-induced HaCaT cells for 48 h were significantly increased compared with those in the vehicle group, while the TCs-cocultured group showed decrease in the late apoptosis, early apoptosis and apoptosis ratios compared with the LPS group. For HDMECs, at 24 h, the dead cell ratio in LPS-induced HDMECs was significantly increased compared with the ratio in the vehicle group, while the TCs-cocultured group showed a decreased death ratio compared with the LPS group. The TCs-cocultured group showed a decreased early apoptosis ratio in HDMECs compared with the LPS-induced group, while there was no significant difference between the LPS-induced HDMECs and TCs-coculture groups. The death ratio in LPS-induced HDMECs for 48 h was significantly increased compared with vehicle group, and the late apoptosis and apoptosis ratios decreased in LPS-induced HDMECs. However, the TCs group demonstrated increased death, late apoptosis, early apoptosis and whole apoptosis ratios compared with the LPS-induced group. The death ratio of the LPS-induced L929 for 24 h was significantly increased compared with that in the vehicle group, while the TCs group showed a decreased death ratio compared with the LPS group. TCs co-culture increased the ratios of late apoptosis, early apoptosis and whole apoptosis ratios in LPS-induced L929 cells. The death and late apoptosis ratios were decreased in the LPS-induced L929 for 48 h, and the whole apoptosis ratio was increased in the LPS-induced L929 group compared with vehicle group. TCs co-culture elevated the death ratios and early apoptosis in LPS-induced L929 cells, and there was no difference in the late apoptosis and whole apoptosis ratios in the TCs co-culture or LPS-induced L929 groups (Fig. 7).

### TCs exhibited a strong ability to scavenge ROS in HDMECs and L929 cells induced by LPS

**Fig. 8:**
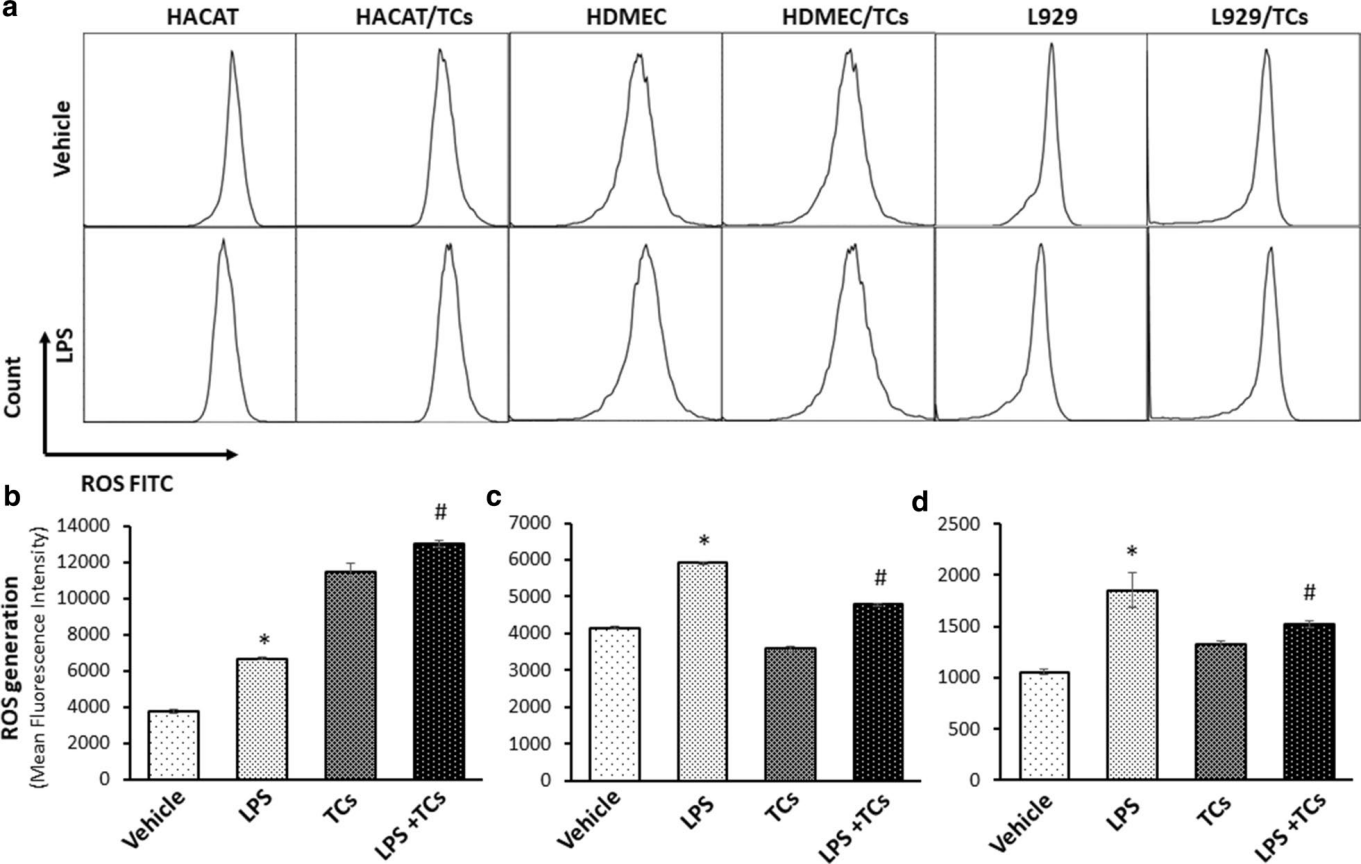
ROS levels were detected in LPS induced HaCaTs, HDMECs and L929 inflammation cell models with TCs treatment by flow. **a** Representative pictures of ROS for LPS stimulated HaCaTs, HDMECs and L929 alone or co-cultured with TCs. **b**–**d** The comparison of ROS contents in LPS stimulated HaCaTs, HDMEC and L929 alone or co-cultured with TCs. ^*^P < 0.05 compared with vehicle; ^#^*P* < 0.05 compared with LPS treated group; ^ns^*P* > 0.05, LPS treated group compared with vehicle or TCs-cocultured group compared with LPS-induced group. Data are expressed as the mean ± SEM

The effects of TCs on ROS in LPS-treated HaCaT cells, HDMECs or L929 cells were further analysed by flow cytometry. The results showed that HaCaT cells, HDMECs or L929 cells induced by LPS exhibited significantly higher ROS than control cells. TCs co-culture significantly decreased LPS-induced ROS in HDMECs and L929 cells. However, TCs did not affect ROS in LPS-induced HaCaT cells, whereas TCs elevated ROS in LPS-induced HaCaT cells (Fig. 8).

### TCs alleviated the inflammation in HaCaT cells, HDMECs and L929 cells induced by LPS

**Fig. 9:**
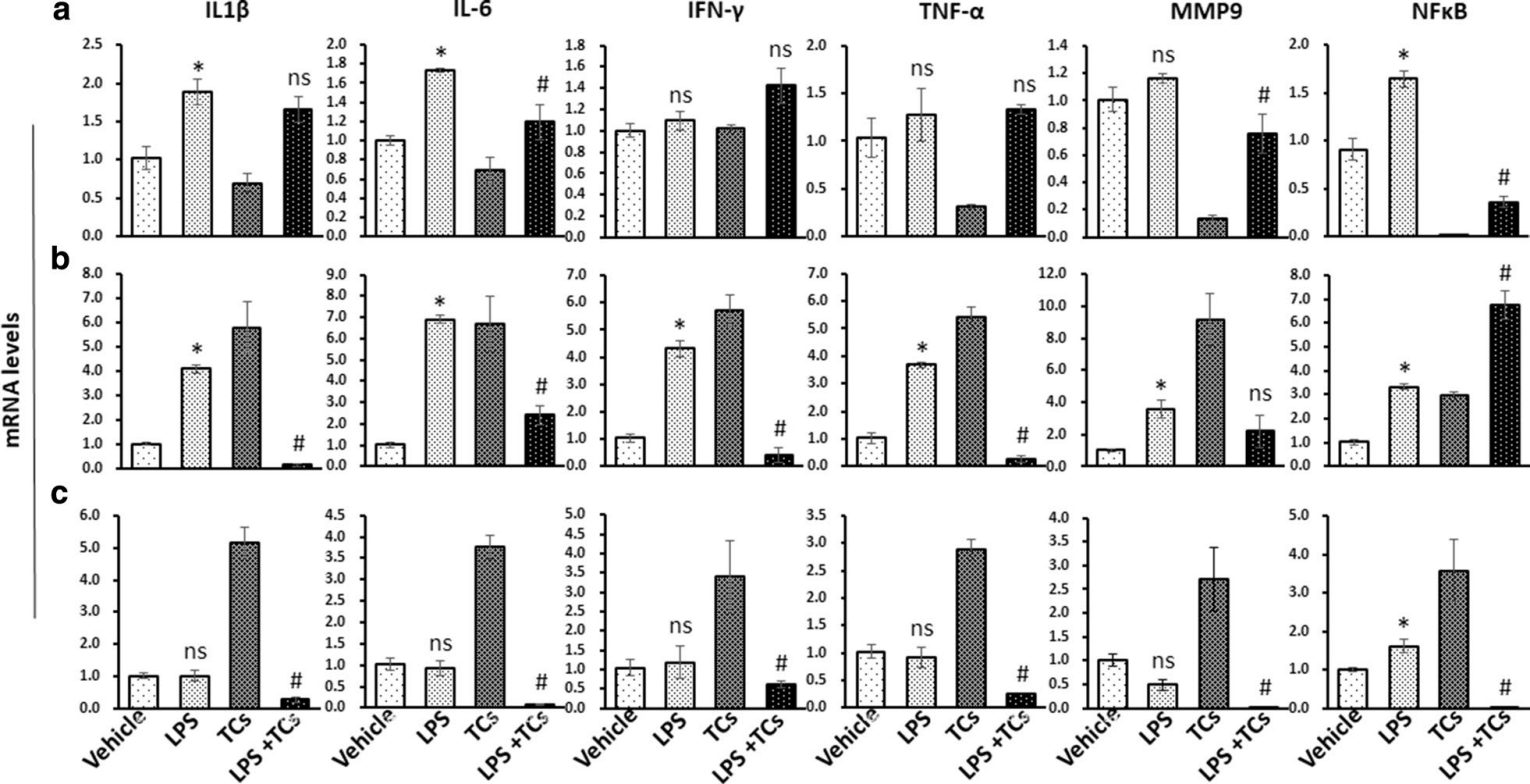
Inflammatory factors mRNA levels were detected in LPS induced HaCaTs, HDMECs and L929 inflammation cell models with TCs treatment. **a** The mRNA level of IL-6, IL1β, IFN-γ, TNF-α, MMP9 and NFκB were detected in LPS induced HaCaTs with or without TCs treatment. **b** The mRNA level of IL-6, IL1β, IFN-γ, TNF-α, MMP9 and NFκB were detected in LPS induced HDMECs with or without TCs treatment. **c** The mRNA level of IL-6, IL1β, IFN-γ, TNF-α, MMP9 and NFκB were detected in LPS induced L929 with or without TCs treatment. ^*^P < 0.05 compared with vehicle; ^#^*P* < 0.05 compared with LPS treated group; ^ns^*P* > 0.05, LPS treated group compared with vehicle or TCs-cocultured group compared with LPS-induced group. Data are expressed as the mean ± SEM

We further detected the levels of inflammatory cytokines in HaCaT cells, HDMECs and L929 cells co-cultured with TCs and induced with LPS. The results showed that IL-6, IL1β and NFκB mRNA levels were upregulated in HaCaT cells after LPS stimulation for 24 h, and TCs reduced the IL-6, IL1β and NFκB mRNA levels induced with LPS (Fig. 9a). The mRNA levels of IL-6, IL1β, IFN-γ, TNF-α, MMP9 and NFκB were increased in HDMECs after LPS stimulation, and TCs reduced the amounts of IL-6, IL1β, IFN-γ, and TNF-α. The mRNA level of MMP9 was not influenced, and the level of NFκB increased with TC co-culture (Fig. 9b). Only NFκB increased in the L929 and LPS groups compared with the Vehicles, and all inflammatory factors decreased in the LPS-stimulated and TCs-treated groups compared with the LPS group (Fig. 9c).

### EGF mRNA level was increase in TCs after LPS stimulation

**Fig. 10:**
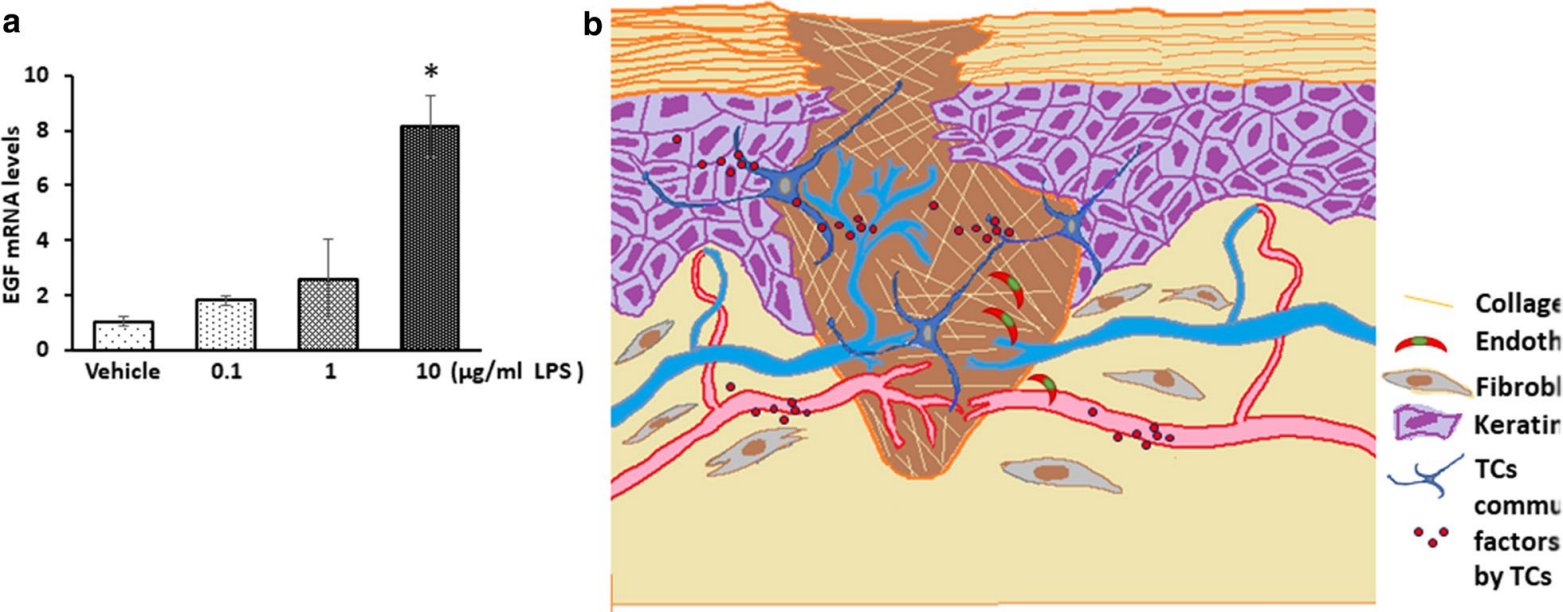
EGF mRNA levels in TCs after LPS stimulation and the Schematic representation of the working model. **a** EGF mRNA levels in TCs after LPS stimulation. *P < 0.05 compared with vehicle. Schematic representation of the working model. **b** Co-culture with TCs lead to up-regulation of migration in HaCaTs and HDMECs, and down-regulation of apoptosis and death ratios of HaCaTs treated with LPS. ROS contents and inflammatory factors mRNA levels were elevated in LPS induced HaCaTs, HDMECs and L929 inflammation cell models with TCs treatment, which might provide the possible mechanism underlying the role of TCs in wound healing

As some groups described, TCs could secret many cytokines, including EGF and VEGF [16, 17]. Since the proliferation of cells cocultured with TCs was altered, EGF was selected as the representative factor and being detected. For this purpose, TCs were incubated with 0.1, 1 or 10 μg/ml LPS for 24 h. Then, RT‐PCR analyses were used to detect the levels of EGF mRNA. Result shows that the mRNA level of EGF were increased after 10 μg/ml LPS stimulation for 24 h (Fig. 10a).

## Discussion

In this study, we found an accumulation of TCs in the granulation tissues of chronic wounds. The transplantation of telocytes reduced the extension of inflammatory cell infiltration in granulation tissues and reversed LPS-induced wound healing delay in mouse skin wound models. An in vitro study showed that TCs might reverse the anti-proliferation, migration and death effects induced in HaCaT cells, HDMECs, and L929 cells. The underlying mechanism may be mediated by LPS via ROS and inflammatory factor inhibition (Figs. 8, 9).

TCs, as a newly discovered stromal cell type, have been found in the skin of newborn rats. TCs were observed to be damaged and to disappear gradually in the affected skin of systemic sclerosis patients, and ultrastructure degeneration was found in psoriatic skin [18]. In our study, the accumulation of PDGFRα^+^ TCs in the dermis layers of chronic wounds (Fig. 1) indicated that PDGFRα^+^ TCs located in human skin and might have functions in chronic skin wounds. Intramyocardial injection of TCs had been found to result in myocardial protection by inducing angiogenesis and alleviating cardiac fibrosis [19]. In the present study, we demonstrated that intradermal injection of a TCs suspension promoted wound healing in LPS-stimulated skin wound probably mediated by relieving inflammatory cell infiltration in LPS-stimulated mouse granulation tissues models (Figs. 3, 4). Wound healing rates and inflammatory cell infiltration ratios showed no difference between vehicle and TCs group, which indicated that TCs need stimulant such as LPS and would have protection roles. As the passenger of injected TCs was PBS, the affection of TCs on LPS-induced wound healing were probably by secreted molecules after injection.

To maintain microenvironmental haemostasis, complex communication with adjacent or distant cells is required for a special location within connecting cells [10]. The types of cells connected to TCs include fibroblasts, epithelia, myocytes, and other organ cells [6]. TCs have been suggested as a type of connecting cell dependent on its telopodes (Tps) and 3D structures, or through secreted mediators, connectors, or ligand receptor for interactions [20]. TCs are known to be involved in organ injury with the ability to promote proliferation and angiogenesis [21]. Our studies demonstrated the effect of TCs on the proliferation, migration and apoptosis of HACAT cells, HDMECs, and L929 cells in the presence of LPS mainly mediated by improving HaCaTs migration and protecting them from LPS-induced apoptosis and cell death. Induced apoptosis in dermal keratinocytes can delay wound healing processes [22]. In our research, TCs non-contact co-culture reduced the LPS induced early, late and whole stages of apoptosis and cell death in HaCaT cells demonstrated their effects independent of Tps. Recently study showed that cytokines, including VEGF and EGF, released by TCs are considered to be key factors were considered to be the mechanism for TCs promotion on angiogenesis [16, 17].

Low concentrations of ROS played a pivotal role in the orchestration of the normal wound-healing response and had been demonstrated to be essential in cell-survival signaling in wound healing [23]. The downstream signalling of ROS, including IDH2 expression associated with cell apoptosis in mouse skin through ROS-dependent ATM-mediated p53 signaling. Our results indicated that TCs could decrease the apoptosis in LPS-induced HDMECs and L929 cells mediated by ROS signalling, however, increase ROS levels were increased in LPS-induced HACAT cells with TCs co-culture (Fig. 7). These results further demonstrated the complexity of function and mechanisms mediated TCs communication with altered cell types. The mechanisms in the function of ROS on apoptosis in target cell might be acting as secondary messengers in recruitment of lymphoid cells to the wound site and effective tissue repair. Moreover, ROS has the ability on angiogenesis regulation in the wound-healing area [24].

Inflammatory factors are deeply involved in LPS-induced apoptosis and injury, and could be regulated by ROS [25]. Secreting IFN-γ to induce the differentiation of naive CD4^+^ T cells into Th1 cells had been considered for TCs to improve allergen-induced airway inflammation and hyper-responsiveness [7]. Our current study demonstrated that TCs reversed LPS-induced IL-6 expression in HaCaT cells, HDMECs, and L929 cells. Moreover, TCs may elevate altered cytokines or inflammatory factors in LPS-induced HaCaT cells, HDMECs, and L929 cells, thus alleviating the inflammatory response seen in wounds. Toll-like receptor 4 (TLR4) is a classical receptor for LPS and induced downstream signaling. LPS strongly induces pro-inflammatory responses and TNF-α production of Mϕ by activating downstream p38 after binding to TLR4/MD-2 receptor complex [26]. LPS probably activate ERK1/2 pathway and JNK pathway and lead to MMP-9 expression [27]. LPS-induced matrix metalloproteinases (MMP)-1, MMP-9 expression in THP-1 cells [28]. Recent study demonstrated that LPS promoted the production of IL-1β and TNF-α in response to infection mediated by a mechanism for A2AR targeting in microglia [29]. LPS-stimulated IL-6 and IL-8 mRNA expression by IL-6 and IL-8 promoter methylation is crucial for the inflammatory response in bovine endometrial cells [30]. These studies indicated that LPS could stimulated altered inflammatory factors mediated by various mechanisms in diverse cell types and further illustrated that individual functions of TCs on various cell types.

In conclusion, the present study first demonstrated that the transplantation of TCs could alleviate inflammatory responses and improve LPS-induced skin wound healing mouse models. Co-culture with TCs led to the upregulation of migration in HaCaT cells and HDMECs and the downregulation of apoptosis and death ratios in HaCaT cells treated with LPS. ROS contents and inflammatory factor mRNA levels were elevated in LPS-induced HaCaTs, HDMECs and L929 inflammatory cell models with TC treatment. Combining the results of the present study with those of previously published studies, we propose that cell–cell communication centred around TCs can improve skin wound healing delay.

However, there are much more questions refer to the mechanisms involved in TCs protection function derived from our current study: (1) the exact factors released by TCs or contained in exosomes produced by TCs; (2) the processes that TCs injection and interact with other type of cells such as epithelial cell during inflammation, whether TCs could lead to or reversing epithelial-mesenchymal transition (EMT) et al., (3) were there some other cell types, like immune cells are the target cells of the cell- cell communication of TCs, and whether the recently identified Toll-like receptors (TLR) 4 and 5/iNOS signaling pathway were involved in this processing [31]? Therefore, the potential bio-functions of TCs in certain pathological conditions in wounds healing and mechanisms of cell–cell communication between TCs and other cells need to be further explored.

## Data Availability

Not applicable.

## Abbreviations

TCs: telocytes
Tps: telopodes
SV40: Simian vacuolating virus 40 small and large T antigen
ANOVA: one‑way analysis of variance
TNF-α: tumor necrosis factor α
LPS: lipopolysaccharide
NF-κB: nuclear factor kappa B
ROS: reactive oxygen species
IL-6: interleukin-6
IL-1β: interleukin-1β
IFNγ: interferon-γ
MMP9: matrix metalloproteinase 9
HDMEC: the human dermal endothelial cells
HaCaT: human keratinocyte
FIB-SEM: focused ion beam scanning electron microscopy
H&E: hematoxylin and eosin
